# Inter-urban mobility via cellular position tracking in the southeast Songliao Basin, Northeast China

**DOI:** 10.1038/s41597-019-0070-1

**Published:** 2019-05-23

**Authors:** Zhanwei Du, Yongjian Yang, Zeynep Ertem, Chao Gao, Liping Huang, Qiuyang Huang, Yuan Bai

**Affiliations:** 1 College of Computer and Information Science, Southwest University, Chongqing, 400715 China; 2 College of Computer Science and Technology, Jilin University, 130012 Changchun, China; 3 Department of Integrative Biology, University of Texas at Austin, Austin, 78705 USA; 4 Department of Statistics and Data Science, University of Texas at Austin, Austin, 78705 USA; 5 School of Public Health, The University of Hong Kong, Hong Kong Special Administrative Region, 999077 China

**Keywords:** Information technology, Civil engineering

## Abstract

Position tracking using cellular phones can provide fine-grained traveling data between and within cities on hourly and daily scales, giving us a feasible way to explore human mobility. However, such fine-grained data are traditionally owned by private companies and is extremely rare to be publicly available even for one city. Here, we present, to the best of our knowledge, the largest inter-city movement dataset using cellular phone logs. Specifically, our data set captures 3-million cellular devices and includes 70 million movements. These movements are measured at hourly intervals and span a week-long duration. Our measurements are from the southeast Sangliao Basin, Northeast China, which span three cities and one country with a collective population of 8 million people. The dynamic, weighted and directed mobility network of inter-urban divisions is released in simple formats, as well as divisions’ GPS coordinates to motivate studies of human interactions within and between cities.

## Abstract (summary)

**Table:** 

Design Type(s)	time series design • source-based data analysis objective • behavioral data analysis objective
Measurement Type(s)	movement quality
Technology Type(s)	digital curation
Factor Type(s)	geographic location • temporal_interval
Sample Characteristic(s)	Homo sapiens • China • populated place

Machine-accessible metadata file describing the reported data (ISA-Tab format)

## Background & Summary

Popular use of cellular phones enables measurements of large-scale human mobility traces, which have become readily available and served as proxy for human mobility. The underlying interactions of meta-populations within and between cities have been extensively studied both in applied work (e.g., inter-urban mobility^1^, urban activities^2^, urban evolution^3^, heterogeneous responses during extreme events^4^), and epidemiology studies of mobility networks^5,6^.

To study human movements, especially among cities, the analytic framework of mobility networks provides a useful way to characterize interactions among people in different sites. Although transportation and interaction patterns between locations change at hourly and daily scales, many studies of human mobility assume they are static^7–9^, neglecting the nature of mobility dynamics. This is, arguably, due to the lack of fine-grained public datasets that could describe the mobility dynamics between cities. There are some open access datasets covering small geographical locations taking into account the time ordering of interactions, such as networks of wifi hotspots within a city^10^ and networks of students in a university campus^11^. However, fine-grained movement datasets covering large geographical regions including multiple cities with large populations are still missing from the open-access datasets.

In this paper, we curate and amass a fine-grained dataset of mobility to study inter-urban interactions. We capture cellular position tracking of millions mobile phone users from an open-data program in Changchun city. Each location in our dataset represents a group of cellular stations in an official administrative division.

We assume that individual stays at a location if her location is the same at least for half an hour in an hour time interval. Directed movement of each individual from a source location *O* to a destination location *D* denotes a change of location for the corresponding individual. We record the time of the directed movement as the time of arriving *D* in our dataset. The overall directed mobility network of locations is finally compiled by sequentially processing the directed movements for all individuals. In the network, a node represents a location. A weighted edge represents the total number of users’ movements between a pair of locations in each hour.

The dataset contains movements of near 3-million anonymized cellular phone users among 167 divisions (henceforth locations), covering 4 geographically adjacent areas (Changchun City, Dehui City, Yushu City, and Nong’an County) for a one-week period starting on August 7, 2017. This total geographic area, located in the southeast Songliao Basin in the center of the Northeast China Plain, Northeast China, covers more than 20 square kilometers and, in 2017, had a population of nearly 8 million.

To facilitate the use of the open data, we process the above raw dataset to extract a dynamic and directed mobility network of locations. We make these networks available through files in CSV file format, separated by commas. There are 2 files released in 2 folders. The first file denotes the mobility network with four columns ordered by origin location, destination location, their weight and time. For spatial analysis applications, we also provide a geospatial file denoting the GPS information for each location, containing three columns ordered by location associated with its latitude and longitude.

Although this described dataset is a major step towards enabling research about human mobility, it has several limitations. First, despite the fact that the dataset covers a cohort of millions of movements, it is only for a one-week period in summer time. Depending on the application, longer periods of time intervals might be needed. Second, we define a user has movement only when s/he stays in a new location at least half an hour. This may also induce bias as it ignores quick movements. Third, the individual’s destination position is the last known recorded location of the individual. This recording might cause bias. The individual might actually already be in D during the whole period of *t* and *t* − 1. Fourth, the individual’s original position might have been unrecorded at an earlier time (e.g., an hour or a day) than her/his recorded arrival time *t*, since it depends on the last time that the user used her/his phone. We caveat the researchers to be careful about their conclusions when using these data.

## Methods

### Original data sources

Our data consist of location records of millions of anonymized cellular phone users for one week starting from August 7, 2017. These locations include 4 geographically neighboring areas (i.e., Changchun City, Dehui City, Yushu City, and Nong’an County). A cellular phone is assumed to be located at the location of the closest cellular base station that it interacts through sending or receiving signals. In the raw movement data each base station is a unique unit. Note that a set of cellular base stations can serve a metapopulation to provide services together.

There are over 12,000 cellular stations with their exact GPS location information. Using the input of GPS positions associated with cellular stations, we can get their official administrative division codes in 2017 version using the Amap APIs (https://lbs.amap.com/api/webservice/guide/api/georegeo), as well as the GPS information of each administrative division. In total, these cellular stations located in 167 divisions, with 100 in Changchun, 27 in Yushu, 18 in Dehui, and 22 in Nong’an. Each division includes 72 stations on average with a standard deviation of 65 stations. We group together a set of base stations as one location if they are within the same division.

There are nearly 3-million phone users in this study. Most of these users are active with enough credits left in their accounts. The accounts with no credit stop receiving signals automatically in a few days by the company’s system. For each user, we aggregate the corresponding location records into hourly movements. Specifically, we assume an individual stays in a location at least half an hour to be considered in that particular location. If a user is spending less than 30 minutes in a location, we assume s/he does not visit the corresponding location during the corresponding hour. Some trips may have large time intervals perhaps due to phones being out of battery power. As such, we do not consider trips whose duration more than 12 hours (less than 0.3% of the total trips). And accordingly, each individual movement from an original location *O* to a destination location *D* at time *t* denotes that an individual moves from *O* and arrive *D* at time *t*. Each location is identified by an anonymized identification code. The demographic information associated with these divisions can be found in the coming official 2018 statistical yearbook (http://cyfd.cnki.com.cn/N2018050240.htm). The telecommunications operator kindly agreed to grant us the rights of sharing these anonymized movement traces and licensing this derived dataset in the framework of the mobility network as Open Data under Attribution 4.0 International (CC BY 4.0) license. We release the raw files of hourly mobility networks in the *figshare* website^12^, as well as initial matlab code (Supplemental File 1) used in this paper.

### Defining the mobility network

Considering each place (a city or a country) as multiple metapopulations in different locations, we construct the directed mobility network for each hour of the week. Each location is represented as a node in our network. Edges are directed, connecting nodes where users move from origins to destinations and weighted by the total number of users’ movements in each hour-location scenario. An individual directed movement from location *i* to location *j* at time *t* denotes that in a user’s movement, location *j* emerges after the previous location *i* at time *t*.

## Data Records

This dataset is released by 2 comma-separated values (CSV) files, each in a folder, including more than 70-million movements^12^. The first file includes the hourly mobility network with four columns ordered by origin location, destination location, edge weight, and arriving time. The weight is the number of movements per hour between the origin location and destination location. The second file includes the GPS information for each location, containing three columns ordered by location associated with its latitude and longitude.

Finally, two folders are used to to group these files^12^. The first folder (Week-Mobility-Network) includes (Mobility.txt), the file of the hourly-mobility network for the entire week. The second folder (GPS-Location) includes (GPS.txt) the file of latitude and longitude information for each location in the mobility network.

1. Mobility.txt In the mobility network, each row represents the total number of hourly movements by people from locations *i* to *j* in the corresponding day. There are four columns ordered by origin location, destination location, their weight, and time. The format for this file is the following.

- Origin: numerical administrative division code for each origin location;
- Destination: numerical identification for each destination location;
- Weight: total number of movements between an origin location and a destination location in the corresponding hour ####-##-##T##+08, following ISO 8601 format (YYYY-MM-DDTHH+08);
- Time: hourly time of arriving at this destination location in ISO 8601 format (YYYY-MM-DDTHH+08), denoting that *W* movements end in the range from T##-1 to T## in ####-##-##. For example, “2017-08-07T09+08’ denotes there are *W* movements arriving destination *D* between 8:00 to 9:00 on 2017-08-07 in the time zone of UTC+8.

(2) GPS.txt The GPS information for each location. The format of this file is organized as three columns ordered by location identifier and the corresponding latitude and longitude information.

- Location: numerical administrative division code for each location;
- Latitude: numerical values for the latitude of the corresponding location;
- Longitude: numerical values for the longitude of the corresponding location.

## Technical Validation

**Fig. 1:**
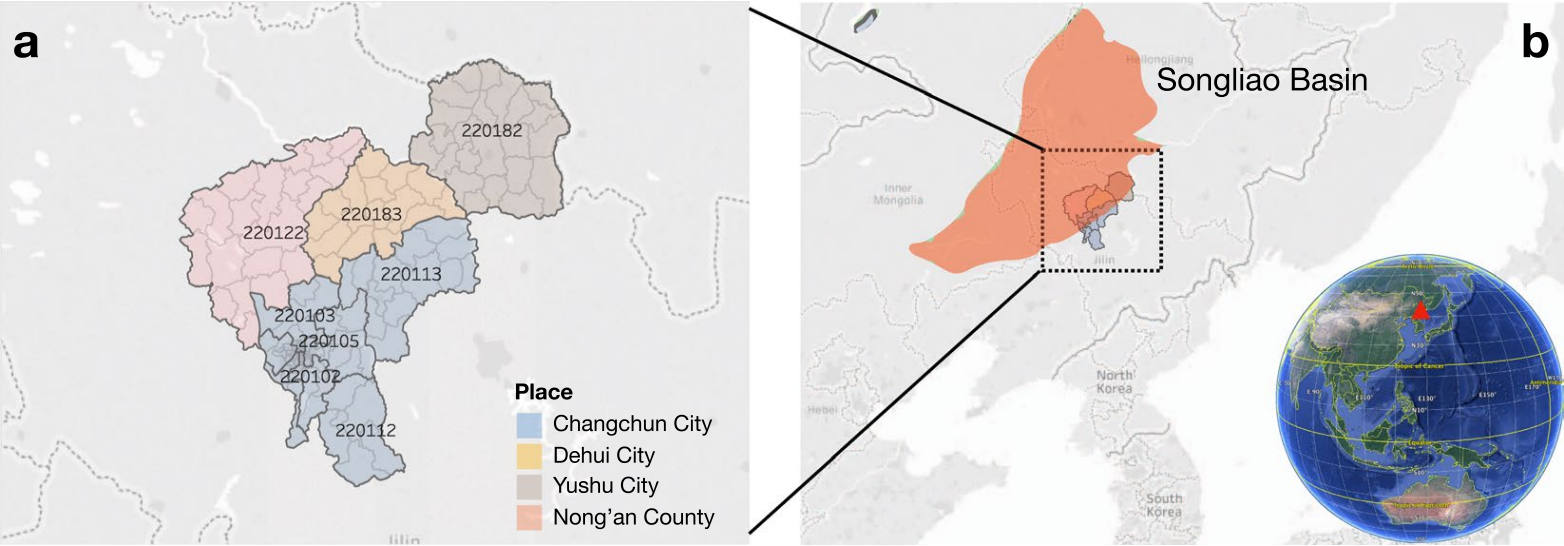
Overview of the geographical distribution of districts. (**a**) The geographical map of 174 divisions across 4 places (Changchun City, Dehui City, Yushu City, and Nong’an County). The number in each region with many divisions denote its administrative division codes in 2017^16^. (**b**) The geographical position of divisions in the earth view, as the southeast part of Songliao Basin, Northeast China. The spatial map was created using OpenStreetMap online platform (http://www.openstreetmap.org/) (© OpenStreetMap contributors) under the license of CC BY-SA (http://www.openstreetmap.org/copyright). More details of the licence can be found in http://creativecommons.org/licenses/by-sa/2.0/. The earth view was created using Google Earth Pro platform with version 7.3 for non-commercial use (https://www.google.com/earth/). Line graphs were drawn using Tableau Software for Desktop version 9.2.15 (https://www.tableau.com/zh-cn/support/releases/9.2.15). The layouts were modified with Keynote version 6.6.2 (http://www.apple.com/keynote/).

The reliability of location and time information of users’ movements in the network data largely depends on the reliability of the underlying source data. We verify the consistency via the geographic-explicit distribution of locations. We visualize 400 locations on a geographic map, as shown in Fig. 1.

### Mobility network

**Fig. 2:**
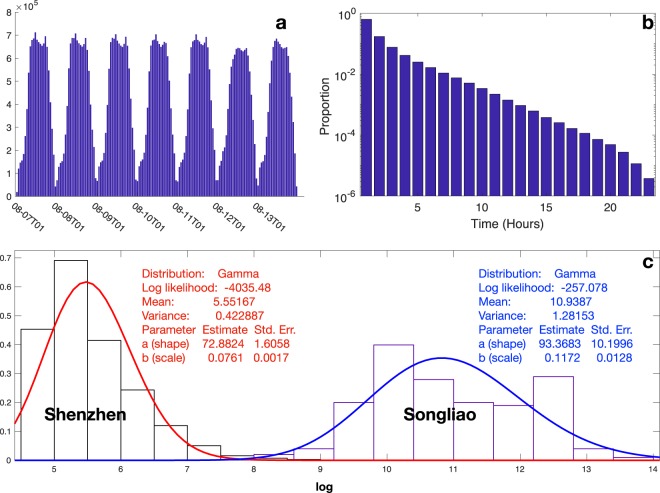
Hourly movement numbers and density plot. (**a**) Hourly movement distribution. The x-axis denotes the 168 hours of the week. The labels in the x-axis follow the pattern of ####-##-##T##+08, following ISO 8601 format (YYYY-MM-DDTHH+08) and inferring the HH hours on YYYY-MM-DD. Y-axis represents the total hourly movement number of the 4 studied places between locations in an hour. (**b**) Trip duration distribution. The x-axis denotes the 24 hours of trip duration. The y-axis denote the proportion of trip number over all across trip durations. (**c**) Empirical d egree distribution with two separate fits: (1) A Gamma distribution for large degree values in the Songliao dataset, (2) A Gamma distribution for small degree values in the dataset of Shenzhen taxi passengers. The x-axis denotes the logarithmic degree. Y-axis is the probability density function for the kernel density estimation. For the mobility network, we estimate the degree of a node as the total number of hourly movements starting or ending in this location across 168 hours in the whole week, as the density plot colored by blue. In contrast, we show the degree distribution of the static mobility network with zones as nodes and passenger flows between nodes as edges, aggregating 2,338,576 trips by taxi passengers in 13,798 taxis in Shenzhen from 18 April 2011 to 26 April 2011 over 1634 zones^7^, as the density plot colored by black. We fit the two datasets by Gamma distributions for our released dataset and Shenzhen. More details of fitness summaries are shown by texts associated with each plot.

In the mobility network, nodes are defined as locations, and edges weighted by the mobility flows between nodes. We verify the consistency of the mobility network with people’s daily life with the hourly movement flows over seven days of the week, as shown in Fig. 2a. A movement denotes an individual movement, whose origin node is different from its destination node. For each hour, we count the number of movements between locations as the hourly movement flow. The hourly movement flows of all working days show two traffic peaks (morning and evening). The morning period is starting at 9:00, and the evening is beginning at 17:00. Both are approximately 4 hours long, similar to the reported mobility flows in the literature for another Chinese city of Shanghai with the morning period starting at 9 am, and the evening period starting 4 pm^2^. As for weekends, traffic peaks are slightly lower and especially weak in the afternoon. Figure 2b shows the trip durations for 24 hours. The y-axis denote the proportion of trip number over all across trip durations. We can observe that trips with less 12 hours account for over 99.7% of the total trips.

The degree of a node denotes the total number of hourly movements passing through the corresponding node during the 168 hours of the week. Figure 2c shows the degree distribution as compared to the degree distribution of another mobility network for another Chinese city (i.e., Shenzen)^7^. We can observe that the part of the log degree distribution for high degree values follows a Gamma distribution with a mean value of 10.9387. In contrast, the reported log degree distribution of the mobility network for Shenzhen^7^ shows a quite different Gamma distribution with a mean value of 5.5516.

### Network structure analysis

**Fig. 3:**
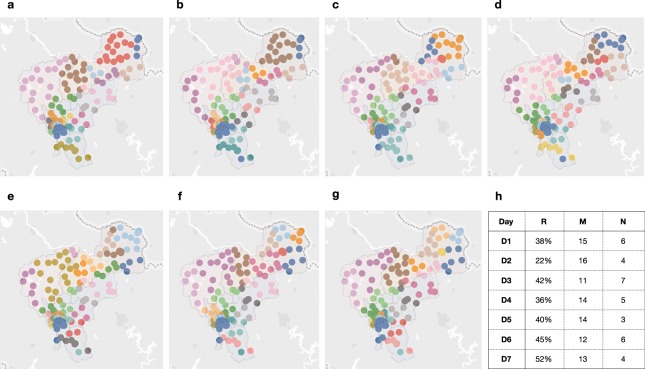
Community structures over days. We construct the daily mobility network via aggregating 24 hourly mobility networks by summing all edges’ weights. The Louvain community detection algorithm^13^ serves to probe community structures based on the daily mobility network for each day of the week (subgraphs a to g). We map community structures with colors denoting different communities in each day. An inter-urban community represents nodes in this community that belong to different locations. We consider 3 community-based measures to reveal the interactions of inter-urban mobility, as shown in subtable h. Specifically, *R* is the percentage of nodes in an inter-urban community over all nodes. *M* denotes the mean number of nodes in a community. *N* represents the number of communities with more than 10 nodes. We can observe Sunday is special, bridging weekday and weekend inter-urban mobility patterns and connect otherwise disconnected inter-urban locations with the highest *R* and the lowest *N*. The spatial map was created using OpenStreetMap online platform (http://www.openstreetmap.org/) (© OpenStreetMap contributors) under the license of CC BY-SA (http://www.openstreetmap.org/copyright). More details of the licence can be found in http://creativecommons.org/licenses/by-sa/2.0/. Line graphs were drawn using Tableau Software for Desktop version 9.2.15 (https://www.tableau.com/zh-cn/support/releases/9.2.15). The layouts were modified with Keynote version 6.6.2 (http://www.apple.com/keynote/).

Additionally, we analyze the community structure of the mobility network using the Louvain community detection algorithm^13^. In each day, the inter-urban mobility network often consists of communities-groups of metapopulations in locations who are highly intra-connected, but only loosely interconnected^14,15^. Figure 3 shows the community structures for each day with colors denoting different detected communities. To explore the interactions of inter-urban mobility, we consider the inter-urban community, which represents nodes in this community belong to different locations. We consider three community-based measures. Specifically, *R* is defined as the percentage of nodes in the community that indicate inter-urban movement. High *R* denotes the strong movement between locations, resulting in multiple inter-urban locations ending up in the same community. *M* denotes the mean size of nodes in a community. High *M* denotes the high average size of locations in a local affiliation. *N* represents the number of communities with more than 10 nodes. High *N* denotes the high variability in mobility with more local affiliations. We can observe Sunday is special with the highest *R* and the lowest *N*, bridging weekday and weekend inter-urban mobility patterns and connect otherwise disconnected inter-urban locations.

## Supplementary Information

### ISA-Tab metadata file

Download metadata file

### Supplementary Information

Supplemental File 1

## Data Availability

Matlab code for data analysis of location correction and mobility network construction can be obtained freely from Supplemental File 1 with no restrictions to access.

## References

[1] Liang X, Zhao J, Dong L, Xu K (2013). Unraveling the origin of exponential law in intra-urban human mobility. Scientific Reports.

[2] Du, Z., Yang, B. & Liu, J. Understanding the spatial and temporal activity patterns of subway mobility flows. Preprint at, https://arxiv.org/abs/1702.02456 (2017).

[3] Lee M, Barbosa H, Youn H, Holme P, Ghoshal G (2017). Morphology of travel routes and the organization of cities. Nature Communications.

[4] Gao C, Liu J (2017). Network-based modeling for characterizing human collective behaviors during extreme events. IEEE Transactions on Systems, Man, and Cybernetics: Systems.

[5] Bai Y (2017). Optimizing sentinel surveillance in temporal network epidemiology. Scientific Reports.

[6] Gao C, Liu J (2013). Modeling and restraining mobile virus propagation. IEEE Transactions on Mobile Computing.

[7] Yan X-Y, Zhao C, Fan Y, Di Z, Wang W-X (2014). Universal predictability of mobility patterns in cities. Journal of The Royal Society Interface.

[8] Simini F, González MC, Maritan A, Barabási A-L (2012). A universal model for mobility and migration patterns. Nature.

[9] Gonzalez MC, Hidalgo CA, Barabasi A-L (2008). Understanding individual human mobility patterns. Nature.

[10] Lenczner, M. & Hoen, A. G. CRAWDAD dataset ilesansfil/wifidog (v. 2015-11-06). https://crawdad.org/ilesansfil/wifidog/20151106 (2015).

[11] Madan A, Cebrian M, Moturu S, Farrahi K (2012). Sensing the “health state” of a community. Pervasive Computing.

[12] Du, Z.-W. *et al.* Bai Inter-urban interactions of mobility via cellular position tracking in the southeast Songliao Basin, Northeast China. *figshare*, 10.6084/m9.figshare.c.4226183.v4 (2018). 10.1038/s41597-019-0070-1 PMC6533259 31123268

[13] Blondel VD, Guillaume J-L, Lambiotte R, Lefebvre E (2008). Fast unfolding of communities in large networks. Journal of Statistical Mechanics: Theory and Experiment.

[14] Girvan M, Newman ME (2002). Community structure in social and biological networks. Proceedings of the National Academy of Sciences of the United States of America.

[15] Clauset A, Newman MEJ, Moore C (2004). Finding community structure in very large networks. Physical Review E.

[16] National Bureau of Statistics of the People’s Republic of China Complete Administrative Division Codes of 2017. http://www.stats.gov.cn/tjsj/tjbz/tjyqhdmhcxhfdm/2017 (2018).

